# SIRT1/FOXO3a axis plays an important role in the prevention of mandibular bone loss induced by 1,25(OH)_2_D deficiency

**DOI:** 10.7150/ijbs.48169

**Published:** 2020-08-19

**Authors:** Haiyun Chen, Xiaoqing Hu, Renlei Yang, Guoping Wu, Qian Tan, David Goltzman, Dengshun Miao

**Affiliations:** 1 The Research Center for Aging, Affiliated Friendship Plastic Surgery Hospital of Nanjing Medical University, Nanjing 210029, China.; 2 Department of Burns and Plastic Surgery, The Drum Tower Clinical Medical College, Affiliated Drum Tower Hospital, Nanjing Medical University, Nanjing 210008, China.; 3 Calcium Research Laboratory, McGill University Health Centre and Department of Medicine, McGill University, Montreal, Quebec H3A 1A1, Canada.

**Keywords:** mandibular bone, Sirt1 promoter, mesenchymal stem cells, antioxidant enzymes peroxiredoxin1

## Abstract

It has been reported that 1,25 dihydroxyvitamin D [1,25(OH)_2_D] deficiency leads to the loss of mandibular bone, however the mechanism is unclear. We investigated whether the Sirt1/FOXO3a signaling pathway is involved in this process. Using a 1,25(OH)_2_D deficiency model induced by genetic deletion in mice of 25-hydroxyvitamin D-1α hydroxylase [1α(OH)ase^-/-^ mice]. We first documented a sharp reduction of expression levels of Sirt1 in the 1α(OH)ase^-/-^ mice *in vivo*. Next, we demonstrated dose-dependent upregulation of Sirt1 by treatment with exogenous 1,25(OH)_2_D_3_ *in vitro*. We then identified a functional VDR binding site in the Sirt1 promoter. By crossing Prx1-Sirt1 transgenic mice with 1α(OH)ase^-/-^ mice we demonstrated that the overexpression of Sirt1 in mesenchymal stem cells (MSCs) greatly improved the 1α(OH)ase^-/-^ mandibular bone loss phenotype by increasing osteoblastic bone formation and reducing osteoclastic bone resorption. In mechanistic studies, we showed, in 1α(OH)ase^-/-^ mice, decreases of Sirt1 and FoxO3a, an increase in oxidative stress as reflected by a reduction of the antioxidant enzymes peroxiredoxin1 (Prdx1), SOD1 and SOD2 expression, and an increase of markers for osteocyte senescence and senescence associated secretory phenotypes (SASP), including β-galactosidase (β-gal), p16, p53 and p21. The targeted overexpression of Sirt1 in the 1α(OH)ase^-/-^ mice restored the expression levels of these molecules. Finally, we demonstrated that a Sirt1 agonist can upregulate FOXO3a activity by increasing deacetylation and nuclear translocation. Overall, results from this study support the concept that targeted increases in Sirt1/FOXO3a signaling levels can greatly improve the bone loss caused by 1,25(OH)_2_D deficiency.

## Introduction

Vitamin D plays an essential role in regulating calcium and phosphate metabolism and maintaining a healthy mineralized skeleton. Vitamin D is obtained from sunlight exposure, and/or dietary foods and supplements. Once vitamin D enters the circulation it is converted by 25-hydroxylase in the liver to 25-hydroxyvitamin D [25(OH)D], which is further converted by the 25-hydroxyvitamin D-1α-hydroxylase [1α(OH)ase] in the kidneys and other tissues to the active form, 1,25-dihydroxyvitamin D [1,25(OH)_2_D]. 1,25(OH)_2_D binds to its nuclear vitamin D receptor (VDR) to exert its physiologic functions, which include promotion of intestinal calcium and phosphate absorption, renal tubular calcium reabsorption, and calcium mobilization from bone 1, 2. Vitamin D deficiency is a major global public health problem in all age groups. It is estimated that 1 billion people worldwide have vitamin D deficiency or insufficiency 3. Vitamin D deficiency has been assumed to be an osteoporotic risk factor, and the vitamin D level of 25-hydroxyvitamin D [25(OH)D] has been reported to be negatively correlated with the occurrence of osteoporosis in Western populations 4, 5. Our previous studies, using homozygous knockout of *1α(OH)ase (Cyp27b1)* (in 1α(OH)ase^-/-^ mice) or double homozygous knockout of *1α(OH)ase* and the parathyroid hormone gene (*Pth*) or double homozygous knockout of *1α(OH)ase* and the calcium sensitive receptor gene (*Casr*), demonstrated that endogenous and exogenous 1,25(OH)_2_D could directly stimulate osteoblastic bone formation via the VDR 6-10. Recently we further demonstrated that 1,25(OH)_2_D plays an anti-osteoporotic role via transcriptionally up-regulating expression of Bmi1 and Ezh2 in osseous cells via the VDR, subsequently, inhibiting oxidative stress and DNA damage, inactivating the p16 and p19 signaling pathways, inducing osseous cell senescence and SASP, facilitating osteoblastic bone formation, and inhibiting osteoclastic bone resorption 11, 12. These studies focused on long bone or vertebrae, however, the role and mechanism of 1,25(OH)_2_D in preventing mandibular bone loss 13 is less well investigated.

The bone formation processes in dentin and dental alveolar bone of the mandibles are distinctly regulated in the 1,25(OH)_2_D-deficient state relative to the processes driving formation in trabecular bone in long bones. The trabecular bone volume and osteoblast numbers are markedly increased in long bones 7, 8, 14, but not in mandibles of 1α(OH)ase^-/-^ mice with increased circulating parathyroid hormone 13. Significant associations exist between periodontal health and intake of vitamin D and calcium, and dietary supplementation with calcium and vitamin D may improve periodontal health, increase bone mineral density in the mandible and inhibit alveolar bone resorption 15, 16. Our previous studies had demonstrated that 1,25(OH)_2_D deficiency resulted in mandibular bone loss in mice 13, 17, however, the underlying mechanism is unclear.

Sirt1 is a nicotinamide adenine dinucleotide (NAD^+^)-dependent deacetylase and is considered as a longevity gene 18. Global Sirt1 transgenic mice are protected against the metabolic decline related to aging 19-22. Recent animal studies indicate that longevity-associated Sirt1 may serve as an attractive pharmacological target for the treatment of osteoporosis and other bone related disorders. Pre-clinical studies demonstrated that mice treated with Sirt1 agonists show protection against age-related and post-menopausal models of osteoporosis 23, 24. Conversely, Sirt1 knockout models display low bone mass phenotypes associated with increased bone resorption and decreased bone formation 25, 26. To investigate the role in osteogenesis of Sirt1 in mesenchymal stem cells (MSCs), we constructed a transgenic mouse model by overexpressing Sirt1 in MSCs using Prx1 as a promoter (Sirt1^Tg^) 27, 28. The transcription factor Prx1, a marker of MSCs, is widely used for mesenchymal lineage-specific knockdown or overexpression of target genes 29. The osteogenesis of alveolar bone is also under the control of Prx1 30. Results from our studies showed that overexpression of Sirt1 in MSCs protected against bone loss induced by Bmi1 deficiency not only in long bone, but also in mandibles 27, 28. Pre-treatment with 1,25(OH)_2_D_3_ attenuated ROS-induced damage and MSC dysfunction by up-regulating the expression levels of Sirt1 in MSCs 31. However, it is unclear whether 1,25(OH)_2_D_3_ can regulate Sirt1 expression at a transcriptional level and whether overexpression of Sirt1 in MSCs protects against bone loss in mandibles induced by 1,25(OH)_2_D_3_ deficiency.

To answer the above questions, we examined the effect of 1,25(OH)_2_D deficiency on Sirt1 expression in mandibular tissue. In view of the fact that the vitamin D receptor (VDR), Prx1 and Sirt1 are all expressed in mandibular mesenchyme 28, 32, 33, we generated 1α(OH)ase^-/-^ mice with Sirt1 overexpression in MSCs (Sirt1^Tg^1α(OH)ase^-/-^). We compared mandibular phenotypes of these mice with those of Prx1-driven Sirt1 transgenic (Sirt1^Tg^), 1α(OH)ase^-/-^, and WT mice to assess whether overexpression of Sirt1 in MSCs corrected 1,25(OH)_2_D deficiency-induced mandibular bone loss. We also examined whether the Sirt1 agonist resveratrol promoted osteogenesis of mandibular MSCs by increasing FOXO3a deacetylation and nuclear translocation using *in vitro* treatment with the Sirt1 agonist resveratrol or the Sirt1 inhibitor Ex527 or with FoxO3a knockdown.

## Materials and Methods

### Animals

We used three types of mutant mouse models in this study: (1) Sirt1^Tg^ mice are transgenic mice that express highly elevated Sirt1 under the control of the 2.4kb Prx1 promoter and were generated in our laboratory and genotyped as described previously 28. (2) 1α(OH)ase^-/-^ mice, were generated through the breeding of heterozygous mice, and genotyped as we previously described 34. (3) Sirt1^Tg^1α(OH)ase^-/-^mice were generated by crossing Sirt1^Tg^1α(OH)ase^+/-^ double-mutant mice. All mice were maintained on a C57BL/6J background. All 1α(OH)ase^-/-^ models were fed a high calcium/phosphate (rescue) diet. Six-month-old male wild-type (WT), Sirt1^Tg^, 1α(OH)ase^-/-^ and Sirt1^Tg^/1α(OH)ase^-/-^ littermates were used in this study. All mice used were approved by the Institutional Animal Care and Use Committee of Nanjing Medical University and maintained in the SPF Laboratory Animal Center of Nanjing Medical University.

### Radiography and micro-computed tomography (micro-CT)

Mandibles were removed and dissected free of all soft tissues for radiography and micro-computed tomography (micro-CT) as described 13.

### Histology and histochemistry

Mandibles were removed and histologically processed as described previously 13, 35. Paraffin sections were stained with hematoxylin and eosin (H&E), or histochemically for total collagen, alkaline phosphatase (ALP) activity or tartrate-resistant acid phosphatase (TRAP) activity as previously described 13, 28. The cortical thickness of mandibles corresponding to the apex of the first molar was measured on H&E stained sections.

### Immunohistochemical staining

Immunohistochemical staining was performed for osterix, SOD1, SOD2, FoxO3a, β-galactosidase (β-gal), p16 and p21 using the avidin-biotin-peroxidase complex technique as described 12. Briefly, dewaxed and rehydrated paraffin-embedded sections were incubated with 6% hydrogen peroxide to block endogenous peroxidase activity and then washed in PBS (pH 7.6). The slides were then incubated at 4°C overnight with the primary antibodies to osterix (Abcam), SOD1/2 (Abcam), FOXO3a (Abcam), β-gal (Abcam), p16 (Abcam), and p21 (Santa Cluz). After rinsing with PBS for 15 min, tissues were incubated with secondary antibody (biotinylated goat anti-rabbit IgG and goat anti-mouse IgG, Sigma). Sections were then washed and incubated with the Vectastain Elite ABC reagent (Vector Laboratories) for 30 min. Staining was done using 3,3-diaminobenzidine (2.5mg/ml) followed by counterstaining with Mayer's hematoxylin.

### Human mandible-derived bone marrow mesenchymal stem cells (BM-MSCs) cultures

Human mandible-derived BM-MSCs were donated by Dr. Wen Sun of Jiangsu dental hospital. Detailed written informed consent was obtained from all volunteers in accordance with protocols approved by the Human Subjects Institutional Review Board of Nanjing Medical University (Approval ID 2016115) 28. Human mandible-derived BM-MSCs were cultured in α-MEM containing 10% FBS with 50 μg/ml ascorbic acid and 100nM dexamethasone (Science cell Research Laboratories, Carlsbad, CA, USA) in the absence or presence of 10^-9^-10^-8^M or 10 μM resveratrol and 10 μM Sirt1 inhibitor Ex527 for 5-10 days. Cells were then stained cytochemically for ALP or senescence associated β-galactosidase (SA-β-gal) as previously described 12, or by immunofluorescence staining for FOXO3a, or for ethynyl deoxyuridine (EdU) incorporation assay. For immunofluorescence staining, cells were fixed with 4% paraformaldehyde and blocked in 10% normal goat serum containing 0.5% BSA and 0.1% Triton X-100 for 1 h and then stained overnight with anti-FOXO3a antibody (Cell Signaling Technology, Danvers, MA, USA;1:500) at 4°C. After rinsing with PBS for 15 min, cells were incubated with DyLight594 goat anti-rabbit IgG (Multi Sciences, Nanjing, China) at room temperature. Slides were mounted with mounting medium containing DAPI (Sigma-Aldrich, St. Louis, MO), and images were taken with a fluorescence microscope (Leica, Wetzlar, Germany). The EdU incorporation assay is the gold standard for detecting cell proliferation. EdU can be incorporated into newly synthesized DNA strands during DNA replication, and cell proliferation can then be detected by fluorescence intensity. For the EdU incorporation assay, human mandible-derived BM-MSCs were cultured in α-MEM containing 10% FBS and 50 μM EdU for 2 h at 37 °C before fixation. Detection of EdU was achieved with the Cell-Light EdU Apollo 567 (catalog no. C10310-1; RiboBio), according to the manufacturer's protocol.

### Immunoprecipitation and Western blot analysis

Whole-cell lysates or cell nuclear lysates were prepared from human mandible-derived BM-MSCs. Immunoprecipitation experiments were extracted by using Pierce™ Crosslink Magnetic IP Kit (Thermo Scientific, Waltham, MA, USA) and a Nuclear and Cytoplasmic Protein Extraction Kit (Thermo Scientific, Waltham, MA, USA). An immunoprecipitation assay was performed as recommended by the supplier. Proteins extracted from 2×10^6^ human mandible-derived BM-MSCs was mixed with 1 μg of antibody and prewashed Protein A/G, then incubated overnight. The bound antigens were eluted from the beads by boiling samples for 10 min. Eluted samples were obtained from SDS-PAGE. Immunoblotting was carried out as previously described 36. Proteins were extracted from mandibles as previously 13. Primary antibodies against Sirt1, FOXO3a, acetylated-lysine, Histone H3, and β-actin (Cell Signaling Technology, Danvers, MA, USA) were used. The immunoreactive bands were visualized by ECL chemiluminescence (Amersham) and analyzed by the Scion image Beta 4.02 (Scion, National Institutes of Health).

### ChIP-qPCR

Human mandible-derived BM-MSCs were cultured to perform chromatin immunoprecipitation (ChIP) analyses of VDR recruitment by using an anti-VDR antibody (Abcam) and SimpleChIP^®^ Enzymatic Chromatin IP Kit (Cell Signaling Technology, Danvers, MA, USA). ChIP analyses were performed as recommended by the supplier to identify the VDR recruitment. The primers used to amplify the 153bp fragment of the human FOXO3a gene promoter were 5'-AGCCCGCTTCTACACTCTGA-3' (forward) and 5'-ATTCTACGATCCGTGCCCCA-3' (reverse). The PCR products were electrophoresed on 2% agarose gels, and visualized by ethidium bromide staining.

### RNA isolation and real-time RT-PCR

Total RNA was extracted from the cultured BM-MSCs and mandibles using Trizol reagent (Invitrogen) according to the manufacturer's instructions. Complementary DNA (cDNA) was synthesized using Synthesis SuperMix (Invitrogen). Real-time RT-PCR was carried out using an Agilent Real-time System. Gapdh was amplified at the same time to normalize gene expression. Each experiment was repeated three times to determine relative gene expression differences. The sequence-specific primers of human and mice are displayed in Table 1.

### Construction of promoter-reporter plasmids and dual-luciferase transient expression assay

Analysis of the transcriptional activity of the VDR was conducted. The full coding sequence of VDR was amplified and cloned into a pCDNA3.1 vector used as an effector vector. The promoters of the Sirt1 gene were cloned into the GV238-LUC reporter vector. The plasmid pGL3-Sirt1 containing -agagtaca- in the promoter region of the human Sirt1 gene linked to the promoterless firefly luciferase gene, and a mutational plasmid pGL3-Sirt1-mut in which -agagtaca- was changed into -ctctccat-, were constructed. Human mandible-derived BM-MSCs cells were plated in 24-well Falcon plates at a density of 100,000 cells/well in α-MEM with 10% FBS 24 hours prior to transient transfection. Plasmids were transfected in individual wells using Opti-MEM and liposome according to the manufacturer`s protocol. Briefly, each well was treated with pcDNA3.0-VDR and pGL3-Sirt1 or pGL3-Sirt1-mut (with or without 10^-8^M 1,25(OH)_2_D_3_), along with 40 ng of Renilla reniformis luciferase. The Renilla reniformis luciferase plasmid allows for constitutive, low-level expression to monitor DNA transfection efficiency. Forty-eight hours post-transfection, the cells were lysed in 1× passive lysis buffer (Promega, Madison, WI) and the lysates were collected. Each lysate was then analyzed sequentially for Firefly and Renilla luciferase activity using a Dual-Luciferase Assay Kit (Promega Corp., Madison, WI). All operating procedures followed the instructions provided by the reagent kit. The mean ratio of Firefly/Renilla for 6 biological replicates (wells) was calculated for each experimental treatment group.

### RNA interference and plasmid transfection

Human small interfering RNAs (siRNAs) for FOXO3a and the control siRNAs (siCtrl) were designed and synthesized by Invitrogen (Grand Island, NY, USA). The FOXO3a plasmid and its negative control vectors were purchased from GeneChem (Shanghai, China). Lipofectamine 3000 (L3000015, Invitrogen) and used for transfection according to the manufacturer's instructions.

### Statistical analysis

Results are expressed as mean ± S.E.M. Statistical analysis was performed using GraphPad Prism 5 software (GraphPad Software Inc., San Diego, CA, USA). Comparisons between two groups were analyzed using a two-tailed unpaired Student's *t*-test. Comparisons among three or more groups were performed using two-way ANOVA followed by Dunnett's postdoc multiple comparisons. *P* values <0.05 were considered statistically significant.

## Results

### 1,25(OH)_2_D_3_ up-regulates Sirt1 expression via VDR-mediated transcription

**Figure 1:**
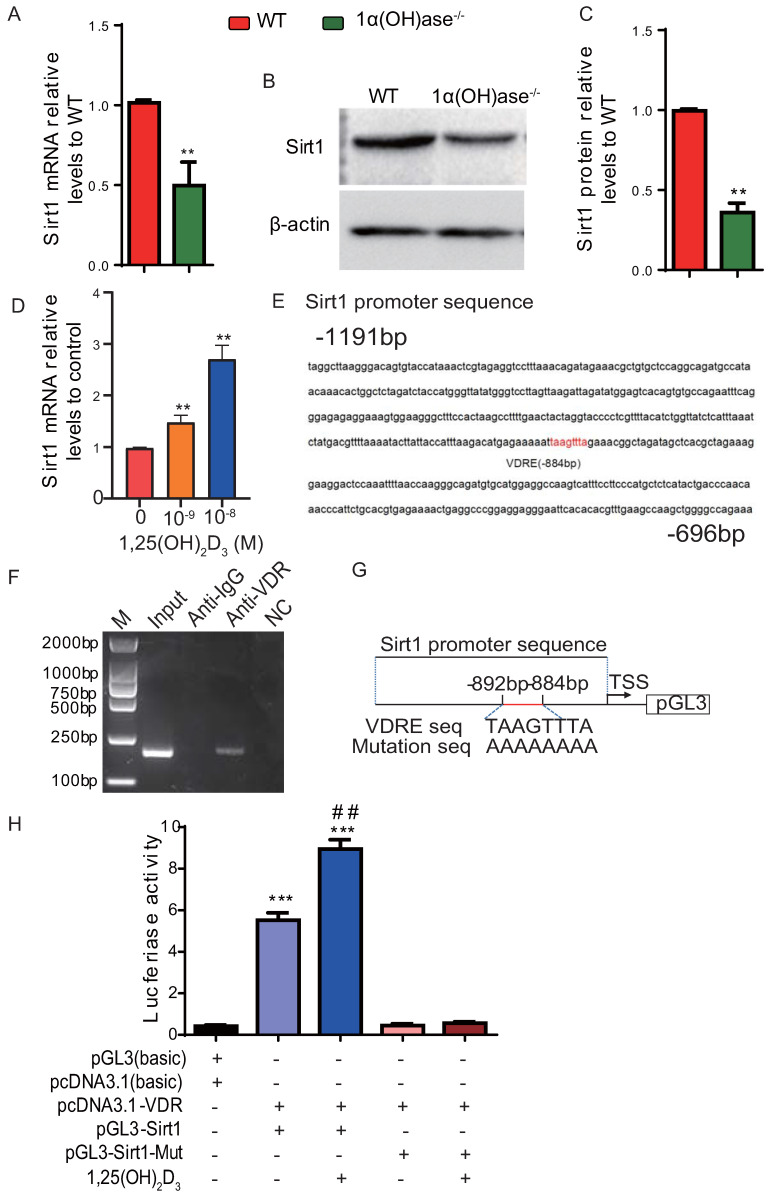
** 1,25(OH)_2_D_3_ up-regulates Sirt1 expression through VDR-mediated transcription.** (**A**) Real-time RT-PCR analysis of mandible tissue extracts from 6-month-old WT and 1α(OH)ase^-/-^ mice showing expression of Sirt1. (**B**) Western blotting was performed on tissue extracts for Sirt1 protein levels and (**C**) the Sirt1 protein relative levels to β-actin were assessed by densitometric analysis. (**D**) mRNA expression levels of Sirt1 in mandible derived BM-MSCs cultured with 10^-9^-10^-8^ M 1,25(OH)_2_D_3_ for 24 hrs, demonstrated by real-time RT-PCR. (**E**) The VDR-like element (-taagttta-) motif in the Sirt1 promoter was detected by bioinformatics analysis. (**F**) ChIP analysis was performed using a negative control immunoglobulin G (IgG) or anti-VDR antibody in human mandible derived BM-MSCs cells. (**G**) Schematic diagram of the structure of the pGL3-*Sirt1* promoter reporter plasmid and of the mutant pGL3-*Sirt1* promoter reporter plasmid. (**H**) *Sirt1* promoter activity was measured by a dual luciferase reporter assay. Human mandible derived BM-MSCs were transfected with an expression plasmid of the human *VDR* (pcDNA3.1) or an empty vector (pcDNA3.1) and transfected with the pGL3-*Sirt1* promoter, a pGL3-*Sirt1* promoter-mutant, or the pGL3 basic vector. A Renilla reniformis luciferase reporter plasmid was co-transfected for 48h. 1,25(OH)_2_D_3_ at 10^-8^ M or vehicle were added following the transfections. Luciferase activity was collected and normalized to Renilla's values at 48 h after transfection. Values are mean ± s. e. m. of 3 determinations per group. *^:^ *P* < 0.05, **^:^ *P* < 0.01 compared with WT or control group; ##^:^ *P* < 0.01 compared with 1,25(OH)_2_D_3_ untreated group.

We previously reported that 1,25(OH)_2_D deficiency in the 1α(OH)ase^-/-^ mice accelerated alveolar bone loss 13, 17. In this study we assessed whether the alveolar bone loss induced by 1,25(OH)_2_D deficiency is mediated via the NAD-dependent deacetylase sirtuin-1 (Sirt1). We first examined the alteration of Sirt1 expression at both mRNA and protein levels in mandibular tissue from wild-type and 1α(OH)ase^-/-^ mice. Our results demonstrated that Sirt1 mRNA and protein expression levels in mandibular tissue were down-regulated dramatically in 1α(OH)ase^-/-^ mice compared with their wild-type littermates (Figs. 1A-C). We then examined the effect of exogenous 1,25(OH)_2_D_3_ on Sirt1 expression in mandible-derived bone marrow-mesenchymal stem cells (BM-MSCs); we found that 1,25(OH)_2_D_3_ significantly up-regulated mRNA expression levels of Sirt1 in a dose-dependent manner in mandible derived BM-MSCs cultured with 10^-9^-10^-8^ M 1,25(OH)_2_D_3_ for 24 hrs (Fig. 1D). To assess whether 1,25(OH)_2_D_3_ regulates Sirt1 via the VDR at a transcriptional level, a VDRE like sequence at position -884 in the 5'-flanking regions of the Sirt1 promoter, retrieved from the NCBI human genome data base, was identified by computer-aided analysis (Fig. 1E). Results of a ChIP-PCR assay demonstrated that the VDR could directly bind to the Sirt1 promoter on the predicted binding site (Fig. 1F). Using human genomic DNA as a template, PCR was used to amplify the whole promoter segment -863 to -1055. The PCR products without and with mutated VDRE were then cloned into pGL3-basic vectors (pGL3-Sirt1, pGL3-Sirt1-mut), which were transiently transfected into mandible derived BM-MSCs (Fig. 1G). Luciferase reporter assays demonstrated that luciferase expression levels were increased significantly in mandible derived BM-MSCs transfected with a Sirt1 binding sequencing plasmid compared with the vehicle, and the luciferase expression levels were augmented by 1,25(OH)_2_D_3_; in contrast, luciferase activity was not increased in mandible derived BM-MSCs transfected with a pGL3-Sirt1 mutant plasmid compared with the vehicle (Fig. 1H). Therefore, 1,25(OH)_2_D_3_ up-regulated Sirt1 expression via VDR-mediated gene transcription.

### Overexpression of Sirt1 in MSCs prevents 1,25(OH)_2_D deficiency-induced alveolar bone loss

**Figure 2:**
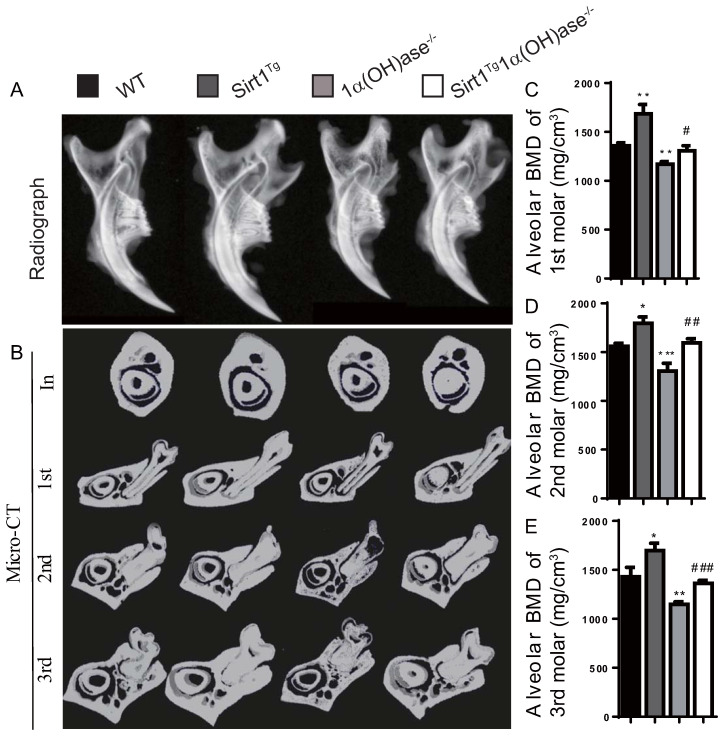
** Overexpression of Sirt1 in MSCs prevents 1,25(OH)_2_D deficiency induced mandibular bone loss.** (**A**) Representative radiographs of mandibles from 6-month-old wild-type (WT), Sirt1^Tg^, 1α(OH)ase^-/-^ and Sirt1^Tg^1α(OH)ase^-/-^ mice. (**B**) Representative images of micro-CT-scanned sections through the incisors in front of the first molar (In), the first (1st), second (2nd) and third (3rd) molars. (**C-E**) Alveolar BMD of 1st, 2nd and 3rd molars. Values are mean ± S.E.M. of 6 determinations per group. *: *P* <0.05, **: *P* < 0.01, ***: *P* < 0.001 compared with WT mice; #: *P* <0.05, ##: *P* <0.01, ###: *P* <0.001 compared with 1α(OH)ase^-/-^ mice.

To investigate whether 1,25(OH)_2_D plays a role in prevention of mandibular bone loss via Sirt1 *in vivo*, we used Prx1-driven Sirt1 transgenic mice (Sirt1^Tg^) and generated a mouse model that overexpressed Sirt1 in MSCs on a 1α(OH)ase^-/-^ background (Sirt1^Tg^1α(OH)ase^-/-^). We then compared their mandibular phenotypes with 1α(OH)ase^-/-^, and wild-type littermates at 6 months of age. By radiography and micro-CT scanning analyses, we found that bone mineral density (BMD) was increased significantly in Sirt1^Tg^ mice, but it was decreased significantly in 1α(OH)ase^-/-^ mice and was not altered in Sirt1^Tg^1α(OH)ase^-/-^ mice at the alveolar bone of 1^st^, 2^nd^ and 3rd molars compared with their wild-type littermates. Nevertheless, it was increased significantly in Sirt1^Tg^1α(OH)ase^-/-^ mice compared with 1α(OH)ase^-/-^ littermates (Figs. 2A-E). These results demonstrated that overexpression of Sirt1 in MSCs could prevent 1,25(OH)_2_D deficiency induced alveolar bone loss *in vivo*.

### Overexpression of Sirt1 in MSCs rescues 1,25(OH)_2_D deficiency-induced alveolar bone turnover defects

**Figure 3:**
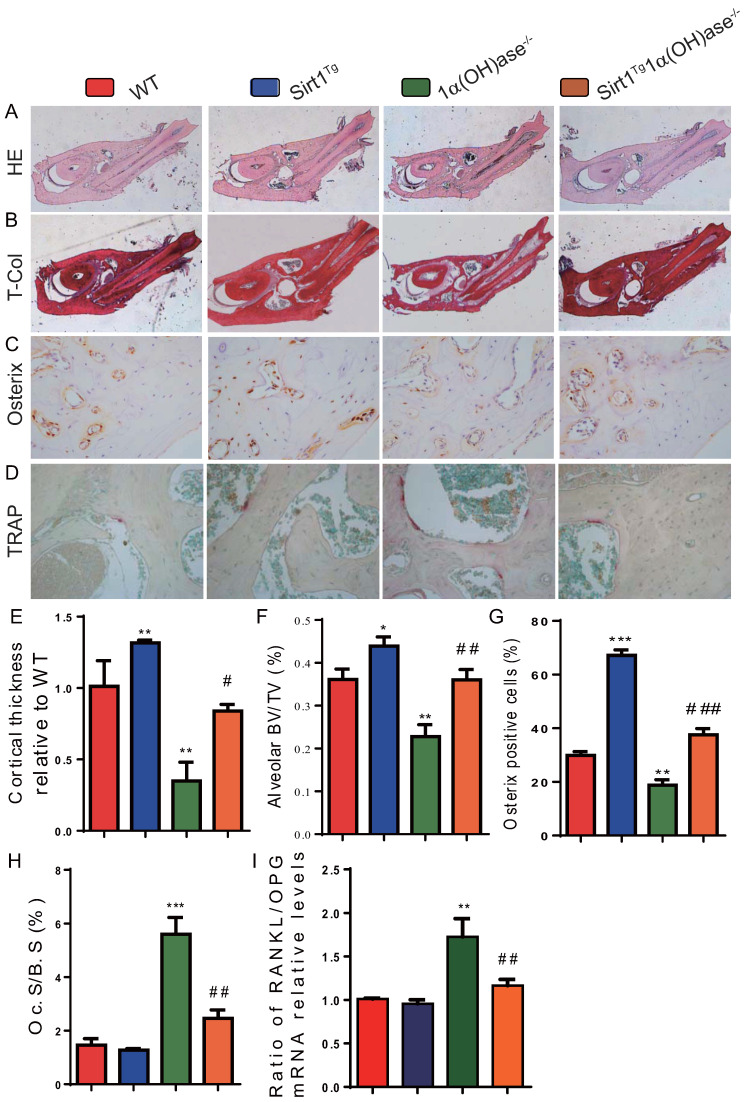
** Overexpression of Sirt1 in MSCs rescues 1,25(OH)_2_D deficiency induced alveolar bone turnover defects.** Representative micrographs of decalcified paraffin-embedded sections through the first molars and the incisors from 6-month-old WT, Sirt1^Tg^, 1α(OH)ase^-/-^ and Sirt1^Tg^1α(OH)ase^-/-^ mice were stained with (**A**) hematoxylin and eosin (HE), (**B**) histochemically for total collagen (T-Col), (**C**) immunohistochemically for osterix and (**D**) histochemically for tartrate-resistant acid phosphatase (TRAP). (**E**) Cortical thickness, (**F**) alveolar bone volume relative to tissue volume (BV/TV, %). (**G**) Osterix-positive cells relative to total cells. (**H**) Osteoclastic surface relative to bone surface (Oc.S/B.S, %). (**I**) RT-PCR of tissue extracts of mandibles for expression of *RANKL* and* OPG*. Messenger RNA expression assessed by real-time RT-PCR is calculated as a ratio relative to *Gapdh*, and expressed as ratio of RANKL/OPG mRNA relative levels to WT mice. Values are mean ± S.E.M. of 6 determinations per group. *: *P* <0.05, **: *P* < 0.01, ***: *P* < 0.001 compared with WT mice; #: *P* <0.05, ##: *P* <0.01, ###: *P* <0.001 compared with 1α(OH)ase^-/-^ mice.

To determine whether 1,25(OH)_2_D deficiency-induced alveolar bone loss prevented by overexpression of Sirt1 in MSCs was associated with alterations of alveolar bone turnover, alveolar bone formation and resorption were examined using histopathological analyses. The results revealed that the cortical bone volume of mandibles, alveolar bone volume and osterix positive cells were increased significantly in Sirt1^Tg^ mice, but were significantly decreased in 1α(OH)ase^-/-^ mice and were not altered in Sirt1^Tg^1α(OH)ase^-/-^ mice compared with their wild-type littermates; however, these parameters were increased significantly in Sirt1^Tg^1α(OH)ase^-/-^ mice compared with 1α(OH)ase^-/-^ littermates (Figs. 3A-C, E-G). In contrast, TRAP positive osteoclastic surface and the ratio of RANKL/OPG mRNA levels were increased dramatically in 1α(OH)ase^-/-^ mice but were not significantly increased in Sirt1^Tg^1α(OH)ase^-/-^ mice compared with their wild-type littermates; these indices were decreased significantly in Sirt1^Tg^1α(OH)ase^-/-^ mice compared with 1α(OH)ase^-/-^ littermates (Figs. 3D, H & I). These results implied that overexpression of Sirt1 in MSCs prevented 1,25(OH)_2_D deficiency induced alveolar bone loss by stimulating osteoblastic alveolar bone formation and inhibiting osteoclastic alveolar bone resorption.

### Overexpression of Sirt1 in MSCs largely corrects 1,25(OH)_2_D deficiency-induced oxidative stress

**Figure 4:**
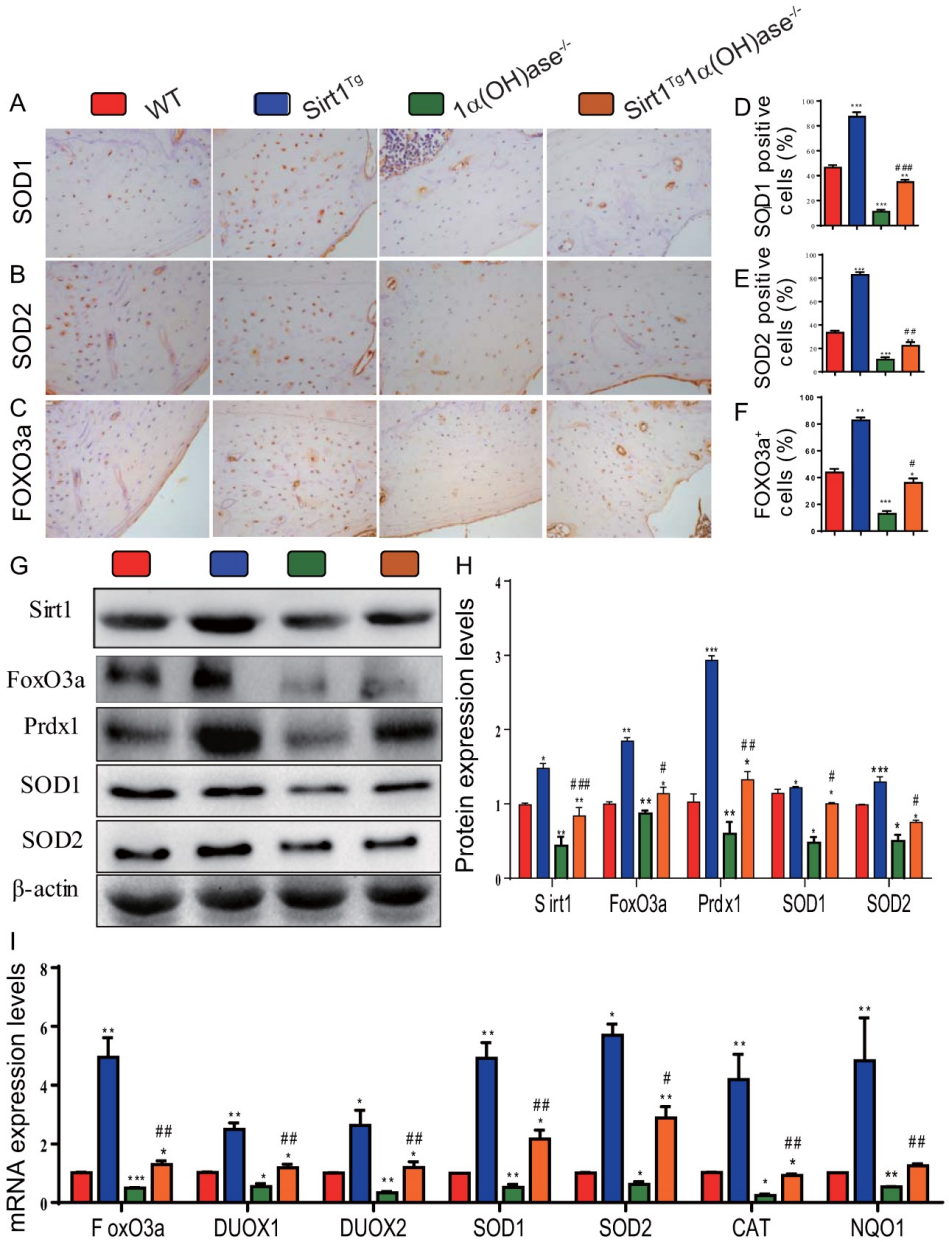
** Overexpression of Sirt1 in MSCs largely corrects 1,25(OH)_2_D deficiency-induced oxidative stress.** Representative micrographs of decalcified paraffin-embedded sections through the first molars and the incisors from 6-month-old WT, Sirt1^Tg^, 1α(OH)ase^-/-^ and Sirt1^Tg^/1α(OH)ase^-/-^ stained immunohistochemically for (**A**) SOD1, (**B**) SOD2 and (**C**) FOXO3a. The percentages of (**D**) SOD1 positive cells, (**E**) SOD2 positive cells and (**F**) FOXO3a positive cells. (**G**) Western blots of mandibular extracts were carried out for expression of Sirt1, FOXO3a, Prdx1, SOD1 and SOD2. (**H**) Protein levels relative to β-actin were assessed by densitometric analysis and expressed as percentage of the levels of WT mice. (**I**) RT-PCR of tissue extracts of mandibles for expression of *FoxO3*,* Duox1*,* Duox2*,* SOD1*,* SOD2*,* CAT* and *NQO1*. Messenger RNA expression assessed by real-time RT-PCR is calculated as a ratio relative to *Gapdh*, and expressed relative to WT mice. Values are mean ± S.E.M. of 6 determinations per group. *: *P* <0.05, **: *P* < 0.01, ***: *P* < 0.001 compared with WT mice; #: *P* <0.05, ##: *P* <0.01, ###: *P* <0.001 compared with 1α(OH)ase^-/-^ mice.

To determine whether 1,25(OH)_2_D deficiency-induced alveolar bone loss prevented by overexpression of Sirt1 in MSCs was associated with alterations of redox balance, we examined the expression levels of antioxidant enzyme proteins and genes in mandibular tissue using immunohistochemistry, Western blots and real-time RT-PCR. The results showed that the percentage of superoxide dismutase (SOD)1, SOD2 and forkhead box O3 (FOXO3a) positive cells (Figs. 4A-F), the protein expression levels of Sirt1, FOXO3a, peroxiredoxin1 (Prdx1), SOD1 and SOD2 (Figs. 4G & H), and the mRNA expression levels of FOXO3a, dual oxidase 1 (Duox1), dual oxidase 2 (Duox2), SOD1, SOD2, catalase (CAT) and quinone oxidoreductase-like protein 1 (NQO1) (Fig. 4I) were increased dramatically in Sirt1^Tg^ mice, but were decreased in 1α(OH)ase^-/-^ mice compared with their wild-type littermates; they were also increased significantly in Sirt1^Tg^1α(OH)ase^-/-^ mice compared with 1α(OH)ase^-/-^ littermates. These results indicate that overexpression of Sirt1 in MSCs largely corrected 1,25(OH)_2_D deficiency-induced oxidative stress by up-regulating the expression levels of antioxidant enzymes.

### Overexpression of Sirt1 in MSCs largely corrects 1,25(OH)_2_D deficiency-induced osteocyte senescence and SASP

**Figure 5:**
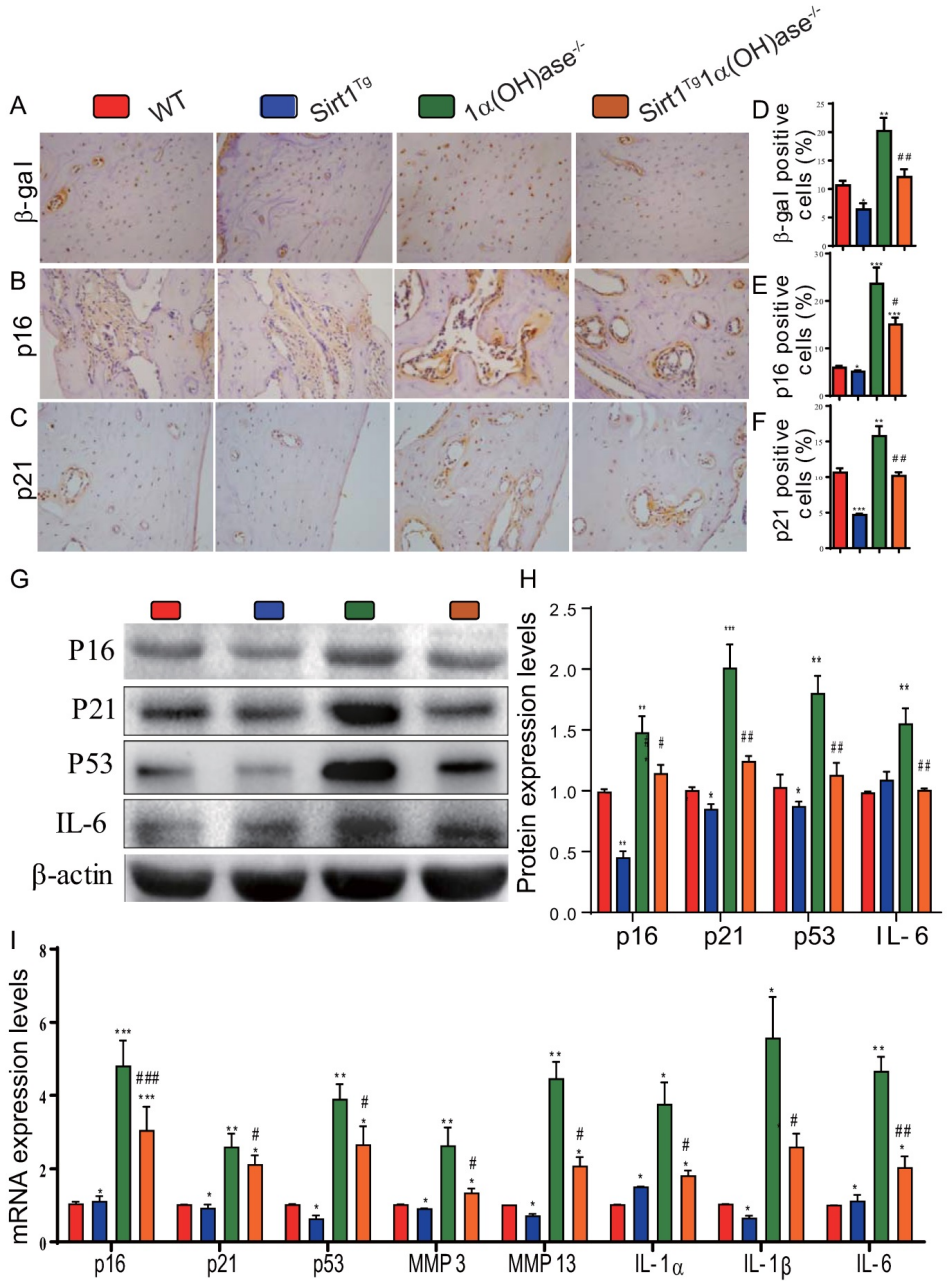
** Overexpression of Sirt1 in MSCs largely corrects 1,25(OH)_2_D deficiency-induced osteocyte senescence and SASP.** Representative micrographs of decalcified paraffin-embedded sections through the first molars and the incisors from 6-month-old WT, Sirt1^Tg^, 1α(OH)ase^-/-^ and Sirt1^Tg^1α(OH)ase^-/-^ stained immunohistochemically for (**A**) β-gal, (**B**) p16, and (**C**) p21. The percentages of (**D**) β-gal positive cells, (**E**) p16 positive cells and (**F**) p21 positive cells. (**G**) Western blots of mandibular extracts were carried out for expression of p16, p21, p53 and IL-6. (**H**) Protein levels relative to β-actin were assessed by densitometric analysis and expressed as percentage of the levels of WT mice. (**I**) RT-PCR of tissue extracts of mandibles for expression of *p16*,* p21*,* p53*,* MMP3*,* MMP13*,* IL-1α*,* IL-1β* and* IL-6*. Messenger RNA expression assessed by real-time RT-PCR is calculated as a ratio relative to *Gapdh*, and expressed relative to WT mice. Values are mean ± S.E.M. of 6 determinations per group. *: *P* <0.05, **: *P* < 0.01, ***: *P* < 0.001 compared with WT mice; #: *P* <0.05, ##: *P* <0.01, ###: *P* <0.001 compared with 1α(OH)ase^-/-^ mice.

To determine whether 1,25(OH)_2_D deficiency-induced alveolar bone loss prevented by overexpression of Sirt1 in MSCs was associated with alterations of osteocyte senescence and senescence associated secretory phenotypes (SASP), we examined the expression levels of cell senescence and SASP related proteins and genes in mandibular tissue using immunohistochemistry, Western blots and real-time RT-PCR. The results showed that the percentage of β-galactosidase (β-gal), p16 and p21 positive osteocytes (Figs. 5A-F), the protein expression levels of p16, p21, p53 and IL-6 (Figs. 5G & H), and the mRNA expression levels of p16, p21, p53, MMP3, MMP13, IL-1α, IL-1β and IL-6 (Fig. 5I) were all increased significantly in 1α(OH)ase^-/-^ mice compared with their wild-type littermates, however, they were decreased significantly in Sirt1^Tg^1α(OH)ase^-/-^ mice compared with 1α(OH)ase^-/-^ littermates. These results indicate that overexpression of Sirt1 in MSCs largely corrected 1,25(OH)_2_D deficiency-induced osteocyte senescence and SASP.

### Sirt1 agonist resveratrol promotes osteogenesis of mandible BM-MSCs by increasing FOXO3a deacetylation and nuclear translocation

**Figure 6:**
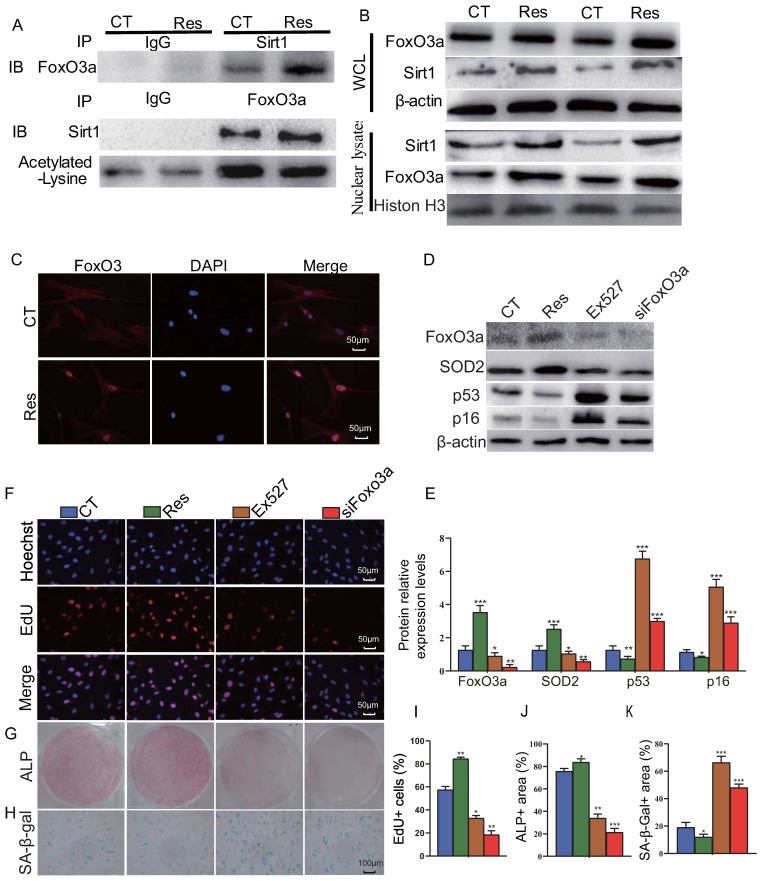
** Sirt1 agonist resveratrol promotes osteogenesis of mandible-derived BM-MSCs by increasing FOXO3a deacetylation and nuclear translocation.** (**A**) Human mandible-derived BM-MSCs were cultured in the absence (CT) or presence of 10μM resveratrol (Res) and whole cell lysates (WCL) were subjected to IP with anti-Sirt1 or anti-FOXO3a antibody and blotted with anti-FOXO3a or anti-Sirt1 or acetylated-lysine antibody. IB, immunoblot. (**B**) Whole cell lysates (WCL) and nuclear lysates were blotted with anti-Sirt1 or anti-FOXO3a antibody. (**C**) Representative micrographs of immunofluorescence staining for FOXO3a (Red), DAPI (Blue) and merge. (**D**) Human mandible-derived BM-MSCs were cultured in the absence (CT) or presence of 10μM resveratrol (Res) or 10 μM Sirt1 inhibitor Ex527 or transfected with siFOXO3a plasmid; whole cell lysates were blotted with anti-FOXO3a or anti-SOD2 or anti-p53 or anti-p16 antibody. (**E**) Protein levels relative to β-actin were assessed by densitometric analysis and expressed as a percentage of the levels of control (CT) cultures. (**F**) Representative micrographs of immunofluorescence staining for Hoechst (Blue), EdU (Red) and merge. Representative images of cells stained cytochemically for (**G**) ALP and (**H**) SA-β-gal. (**I**) The percentages of: EdU-positive cells, (**J**) ALP positive area and (K) SA-β-gal-positive area. Values are mean ± S.E.M. of 3 determinations per group. *: *P* <0.05, **: *P* < 0.01, ***: *P* < 0.001 compared with control (CT) cultures.

To further examine the mechanism of Sirt1 action, human mandible-derived BM-MSCs were treated with 10 μM resveratrol for 6 hours, and then whole cell lysates or nuclear lysates were immunoprecipitated with anti-Sirt1 or anti-FOXO3a antibodies or IgG control and blotted with anti-FOXO3a or anti-Sirt1 antibodies or anti-acetylated lysine antibody. The subcellular localization of FOXO3a was detected by immunofluorescence staining. The results of co-immunoprecipitation studies showed that Sirt1 and FOXO3a were able to bind each other, while resveratrol could enhance their binding, and reduce significantly the acetylation level of FOXO3a (Fig. 6A). Results from Western blots also showed that resveratrol treatment could up-regulate the expression levels of Sirt1 and FOXO3a in total cell protein and more dramatically in nuclear protein (Fig. 6B). Immunofluorescence staining revealed that resveratrol treatment significantly increased the nuclear localization of FOXO3a (Fig. 6C). To further explore the role of Sirt1 to mediate the osteogenesis of BM-MSCs via FOXO3a, we used 10 μM resveratrol and 10 μM Sirt1 inhibitor Ex527 to treat human mandible-derived BM-MSCs, or knocked down FOXO3a in these cells. Alterations of BM-MSC proliferation, osteogenic differentiation and senescence were then analyzed by EdU incorporation, and cytochemical staining for ALP or SA-β-gal respectively. The results showed that resveratrol significantly up-regulated FOXO3a and SOD2 expression levels and significantly down-regulated p16 and p53 expression levels, while both Ex527 and FOXO3a knockdown significantly down-regulated FOXO3a and SOD2 expression levels, and significantly up-regulated p16 and p53 expression levels (Figs. 6D & E). The percentage of EdU-positive cells and the areas of ​​ALP-positive cells were increased significantly in the resveratrol-treated group, decreased significantly in the Ex527-treated group, and decreased more significantly in the FOXO3a knockdown group (Figs. 6F, G, I & J). In contrast, the areas of SA-β-gal-positive cells were decreased significantly in the resveratrol-treated group, increased significantly in the Ex527-treated group and the FOXO3a knockdown group (Figs. 6H & K). These results indicate that the Sirt1 agonist resveratrol can increase the interaction between Sirt1 and FOXO3a, reduce the level of FOXO3a acetylation, and promote its nuclear translocation, thereby promoting the proliferation and osteogenic differentiation of BM-MSCs and inhibiting their senescence.

## Discussion

Our previous studies had demonstrated that 1,25(OH)_2_D deficiency resulted in bone loss in mouse mandibles 13, 17. Other studies have reported that pre-treatment with 1,25(OH)_2_D_3_ attenuated the ROS-induced damage and MSC dysfunction by up-regulating the expression levels of Sirt1 in MSCs 31. In this study, we investigated the mechanism whereby Sirt1 mediated mandibular bone loss induced by 1,25(OH)_2_D deficiency. We found that the expression of Sirt1 at both mRNA and protein levels was remarkably downregulated in mandibular tissue of 1α(OH)ase^-/-^ mice compared with that of wild-type littermates. We also demonstrated that the mRNA levels of Sirt1 were up-regulated in mandible-derived BM-MSCs from wild-type mice when exposed to 1,25(OH)_2_D_3_ in a dose dependent manner. Next, using bioinformatic analysis, ChIP, and dual luciferase assay, we showed that 1,25(OH)_2_D_3_ could regulate Sirt1 expression directly via the VDR at a transcriptional level. These results therefore imply that 1,25(OH)_2_D up-regulates Sirt1 expression in mandible derived BM-MSCs through VDR-mediated transcription.

Previously studies have shown that Sirt1 haploinsufficient female mice exhibited substantially reduced bone formation and mass with reduced osteogenesis 25 and MSC specific deletion of Sirt1 resulted in lower cortical and trabecular bone volume with reduced MSC osteogenesis 26. Recently we generated transgenic mice overexpressing Sirt1 in MSCs driven by the *Prx1* gene and demonstrated that overexpression of Sirt1 in MSCs stimulated osteogenesis of both long bone- and mandible-derived BM-MSCs and increased both long bone and alveolar bone volume 27, 28. Furthermore, overexpression of Sirt1 in MSCs protected against long bone loss induced by Bmi1 deficiency and alveolar bone loss induced by Bmi1 or estrogen deficiency 27, 28. In our current study, we generated 1α(OH)ase^-/-^ mice with Sirt1 overexpression in MSCs. We found that Sirt1 constitutively expressed in MSCs can produce normalization of many of the parameters that were altered in mandibles at 6 months of age in 1α(OH)ase^-/-^ mice, and therefore corrected the mandibular bone loss phenotype caused by 1,25(OH)_2_D_3_ deficiency by increasing mandibular BMD and alveolar bone mass through enhancing osteoblastic bone formation and reducing osteoclastic bone resorption. This indicates that providing Sirt1, a downstream target of 1,25(OH)_2_D, can bypass the requirement for 1,25(OH)_2_D/VDR and normalize the mandibular phenotype of 1,25(OH)_2_D deficiency.

Oxidative stress levels of osteon structures are increased with age 37 and mouse models of osteoporosis are usually accompanied by increased oxidative stress 38, 39. 1,25(OH)_2_D_3_ reduced oxidative stress in human prostate epithelial cells 40 and human endothelial cells by activation of Nrf2-antioxidant signals 41. Cellular senescence is a process in which a cell enters permanent cell cycle arrest, and senescent cells acquire a senescence-associated secretory phenotype (SASP) 42. SASP includes pro-inflammatory cytokines, growth factors, chemokines, and matrix remodeling enzymes 43. Senescent cells cause or aggravate the development of aging-related diseases through their growth arrest phenotype and SASP factors. 1,25(OH)_2_D_3_ has been reported to delay cellular senescence with increased nuclear translocation of VDR in human BM-MSCs 44. We recently demonstrated that 1,25(OH)_2_D_3_ plays an anti-aging role by suppressing oxidative stress, DNA damage, p16-Rb and p53-p21 pathways, and cell senescence and SASP via upregulating Nrf2 34. We also demonstrated that 1,25(OH)_2_D deficiency accelerated age-related osteoporosis by down-regulating Bmi1 and Ezh2 via VDR-mediated transcription, decreasing H3K27me3 and enhancing p16 and p19 transcription, thus inhibiting the proliferation and osteogenesis of BM-MSCs and osteoblastic bone formation, and inducing osteocyte senescence, SASP and osteoclastic bone resorption. In contrast, supplementation of exogenous 1,25(OH)_2_D_3_, overexpression of Bmi1 in MSCs and the deletion of p16 corrected the osteoporotic phenotype caused by 1,25(OH)_2_D deficiency 11, 12. In the current study, we demonstrated that 1,25(OH)_2_D deficiency induced oxidative stress, cellular senescence and SASP in mandibular tissue, by reducing the percentage of SOD1/2 and FOXO3a positive cells, the protein expression levels of Sirt1, FOXO3a, Prdx1 and SOD1/2 and the mRNA expression levels of FOXO3a, Duox1/2, SOD1/2, CAT and NQO1; significant augmentation of the percentage of β-gal, p16 and p21 positive osteocytes, the protein expression levels of p16, p21, p53 and IL-6, and the mRNA expression levels of p16, p21, p53, MMP3, MMP13, IL-1α, IL-1β and IL-6, were also observed. In contrast, these abnormalities induced by 1,25(OH)_2_D deficiency were largely corrected through Sirt1 overexpression in MSCs. Therefore, our results indicate that 1,25(OH)_2_D can prevent mandibular bone loss by inhibiting oxidative stress, cellular senescence and SASP via Sirt1 mediation.

Previous studies have reported that Sirt1 caused the deacetylation of histones at the Sost promoter, thus repressing Sost expression and preventing its negative regulation of osteoblast bone anabolic activity. In addition, Sirt1 promotes the activation of Runx2 and suppression of NF-κB signaling, thereby both stimulating osteoblastogenesis and inhibiting osteoclastogenesis 45-47. It has also been demonstrated that Sirt1 deacetylated FOXO3a resulting in activation of transcriptional activity of FOXO3a and increased expression of its downstream targets, thus regulating cell proliferation, anti-oxidation and apoptosis 48-50. We previously demonstrated that Sirt1 can deacetylate FOXO3a and up-regulate SOD2 expression, thereby inhibiting oxidative stress in bony tissue, inhibiting the senescence of BM-MSCs and stimulating the differentiation of long bone derived BM-MSCs into osteoblasts 27. In the current study, we examined possible mechanisms of Sirt1 in stimulating osteogenesis of mandible derived BM-MSCs by the treatment of Sirt1 agonist resveratrol in cultures *in vitro*. Our results revealed that resveratrol not only up-regulated Sirt1 and FOXO3a expression in total protein and more dramatically in nuclear protein, but also increased the interaction between Sirt1 and FOXO3a, reduced the level of FOXO3a acetylation, and promoted its nuclear translocation. Furthermore, resveratrol promoted the proliferation and osteogenic differentiation of human mandible BM-MSCs and inhibited their senescence, whereas both Sirt1 inhibitor Ex527 and FOXO3a knockdown blocked the above actions of resveratrol. Our results also showed that resveratrol up-regulated the FOXO3a target gene SOD2, down-regulated p53 and p16 expression, whereas Sirt1 inhibitor Ex527 and FOXO3a knockdown down-regulated SOD2 expression, but up-regulated p53 and p16 expression. Previous studies have also shown that the expression of FOXO3a induced significant downregulation of p53 and p16 51; the precise mechanisms whereby FOXO3a regulated p53 and p16, however, remains to be investigated. Our results therefore indicate that the Sirt1 agonist resveratrol can increase the interaction between Sirt1 and FOXO3a, reduce the level of FOXO3a acetylation, and promote its nuclear translocation, and down-regulation of p53 and p16, thereby promoting the proliferation and osteogenic differentiation of BM-MSCs and inhibiting their senescence.

Overall, therefore, the results of this study provide a model that suggests that 1,25(OH)_2_D deficiency induced mandibular bone loss by down-regulating Sirt1 expression through VDR-mediated transcription, increasing FOXO3a acetylation and oxidative stress, activating p16 and p53 signaling, inducing cellular senescence and SASP, and inhibiting osteogenic differentiation of mandibular BM-MSCs with decreased osteoblastic bone formation, and increased osteoclastic bone resorption; These processes can be rescued by overexpression of Sirt1 in MSCs. Our findings have potential translational significance, in that modification of MSCs genetically to overexpress Sirt1, or administration of resveratrol to activate Sirt1, might be novel approaches for the treatment of mandibular bone loss.

## Figures and Tables

**Table 1:** Primers used in this study for real time RT-PCR

Name	S/AS	sequence	species	Tm (°C)	Length (bp)
P53	S	GTCACAGCACATGACGGAGG	mouse	60	129
	AS	TCTTCCAGATGCTCGGGATAC			
P21	S	CCTGGTGATGTCCGACCTG	mouse	58	103
	AS	CCATGAGCGCATCGCAATC			
P16	S	CGCAGGTTCTTGGTCACTGT	mouse	60	127
	AS	TGTTCACGAAAGCCAGAGCG			
IL-1a	S	CGAAGACTACAGTTCTGCCATT	mouse	58	126
	AS	GACGTTTCAGAGGTTCTCAGAG			
IL-1b	S	GCAACTGTTCCTGAACTCAACT	mouse	60	89
	AS	ATCTTTTGGGGTCCGTCAACT			
MMP3	S	ACATGGAGACTTTGTCCCTTTTG	mouse	60	192
	AS	TTGGCTGAGTGGTAGAGTCCC			
MMP13	S	CTTCTTCTTGTTGAGCTGGACTC	mouse	60	173
	AS	CTGTGGAGGTCACTGTAGACT			
IL-6	S	TGTATGAACAACGATGATGCACTT	mouse	60	197
	AS	ACTCTGGCTTTGTCTTTCTTGTTATCT			
SOD1	S	GGTGAACCAGTTGTGTTGTC	mouse	57	203
	AS	CCGTCCTTTCCAGCAGTC			
SOD2	S	GACCTGCCTTACGACTATG	mouse	55	166
	AS	GAAGAGCGACCTGAGTTG			
FOXO3a	S	CTGGGGGAACCTGTCCTATG	mouse	60	210
	AS	TCATTCTGAACGCGCATGAAG			
Duox1	S	AAAACACCAGGAACGGATTGT	mouse	60	123
	AS	AGAAGACATTGGGCTGTAGGG			
Duox2	S	AAGTTCAAGCAGTACAAGCGAT	mouse	60	104
	AS	TAGGCACGGTCTGCAAACAG			
NQO1	S	AGGATGGGAGGTACTCGAATC	mouse	58	144
	AS	AGGCGTCCTTCCTTATATGCTA			
CAT1	S	CAGGTGCGGACATTCTAC	mouse	55	202
	AS	TTGCGTTCTTAGGCTTCTC			
GAPDH	S	CTTGCCAGACACAGATGATCG	mouse	60	163
	AS	GGGGACAGAAGTTGAGTTTC			
VDR-Sirt1-chip	S	TATGGAGTCACAGTGTGCCAG	human	60	192
	AS	GCGTGAGCTATCTAGCCGT			
